# Patterns of *Mycobacterium tuberculosis*-complex excretion and characterization of super-shedders in naturally-infected wild boar and red deer

**DOI:** 10.1186/s13567-015-0270-4

**Published:** 2015-10-30

**Authors:** Nuno Santos, Virgílio Almeida, Christian Gortázar, Margarida Correia-Neves

**Affiliations:** Life and Health Sciences Research Institute (ICVS), School of Health Sciences, University of Minho, Braga, Portugal; ICVS/3B’s, PT Government Associate Laboratory, Braga, Portugal; Centro de Investigação Interdisciplinar em Sanidade Animal (CIISA), Faculdade de Medicina Veterinária, Universidade de Lisboa (FMV-ULisboa), Lisbon, Portugal; SaBio (Health and Biotechnology), IREC, National Wildlife Research Institute (CSIC-UCLM-JCCM), Ciudad Real, Spain

## Abstract

Wild boar (*Sus scrofa*) and red deer (*Cervus elaphus*) are the main maintenance hosts for bovine tuberculosis (bTB) in continental Europe. Understanding *Mycobacterium tuberculosis* complex (MTC) excretion routes is crucial to define strategies to control bTB in free-ranging populations, nevertheless available information is scarce. Aiming at filling this gap, four different MTC excretion routes (oronasal, bronchial-alveolar, fecal and urinary) were investigated by molecular methods in naturally infected hunter-harvested wild boar and red deer. In addition MTC concentrations were estimated by the Most Probable Number method. MTC DNA was amplified in all types of excretion routes. MTC DNA was amplified in at least one excretion route from 83.0% (CI_95_ 70.8–90.8) of wild ungulates with bTB-like lesions. Oronasal or bronchial-alveolar shedding were detected with higher frequency than fecal shedding (*p* < 0.001). The majority of shedders yielded MTC concentrations <10^3^ CFU/g or mL. However, from those ungulates from which oronasal, bronchial-alveolar and fecal samples were available, 28.2% of wild boar (CI_95_ 16.6–43.8) and 35.7% of red deer (CI_95_ 16.3–61.2) yielded MTC concentrations >10^3^ CFU/g or mL (referred here as super-shedders). Red deer have a significantly higher risk of being super-shedders compared to wild boar (OR = 11.8, CI_95_ 2.3–60.2). The existence of super-shedders among the naturally infected population of wild boar and red deer is thus reported here for the first time and MTC DNA concentrations greater than the minimum infective doses were estimated in excretion samples from both species.

## Introduction

Bovine tuberculosis (bTB) is a zoonotic disease whose natural hosts are wild and domestic mammals. Bovine tuberculosis is a disease of economic and public health relevance subjected to eradication programs on livestock in many countries, usually based on test and slaughter and abattoir surveillance strategies [1]. The existence of wildlife reservoirs has been shown to hinder such eradication programs in cattle, as reported to occur with possums (*Trichosurus vulpecula*) in New Zealand, Eurasian badgers (*Meles meles*) in the United Kingdom and Ireland and cervids in North America [2]. In several regions throughout continental Europe bTB is maintained in a multi-host-pathogen system, with *Mycobacterium bovis* and *Mycobacterium caprae* circulating between sympatric wild ungulates (mostly wild boar *Sus scrofa* and red deer *Cervus elaphus*) and free-ranging domestic ungulates (cattle, goats, sheep and pigs) [3]. The wild boar has been shown to act as a maintenance host for bTB in Iberian Peninsula [4]. The red deer is also considered as part of the bTB maintenance community in France [5], Spain [6] and Austria [7]. Wildlife bTB is increasing its host range, geographical distribution and/or frequency of occurrence in several countries and so is considered an emerging disease in Europe [3].

In order to control bTB in wildlife it is essential to gather deep knowledge on factors that affect the intra- and inter-specific transmission of infection, both individually and at a population level. As part of this essential information are the routes of infection, pathology (structure and anatomical location of lesions), routes and levels of excretion and minimum infective doses [2]. While pathology of bTB has been thoroughly documented in many wildlife host species [e.g. 8–10], routes of infection have not been demonstrated but only presumed based upon the location of the lesions [e.g. [8] for wild boar, [11] for red deer, [12] for badgers]. In addition, information on the minimum infective doses have been determined by experimental infections [e.g. [13] for red deer and [14] for wild boar] while data on *Mycobacterium tuberculosis* complex (MTC) excretion is notably scarce. The only study addressing *M. bovis* routes of excretion from naturally infected wild ungulates was performed by Lugton et al. [15], who detected excretion by several routes from red deer: oral (4/53 oropharyngeal swabs), nasal (1/53 nasal swabs), tracheal (1/53 tracheal swabs) and rectal (1/53 fecal samples). Urinary excretion was also investigated but not detected by these authors.

In experimentally infected white-tailed deer (*Odocoileus virginianus*) excretion was shown to occur sporadically by the oral route for up to 90 days post infection (dpi), by the nasal route for up to 85 dpi and it was not detected by the fecal route [16, 17]. Using experimental infection with high doses of *M. bovis,* Palmer and collaborators [18] detected excretion for up to 113 dpi by oral, nasal and fecal routes. In this same study, naïve deer in contact with the experimentally infected animals showed excretion by the oral and nasal routes for up to 90 days post-contact. In some studies shedding has been inferred based on the location and structure of the lesions [8, 10], but *M. bovis* has been cultured from the feces of calves that only presented lesions in the lungs and cephalic-thoracic lymphoid tissues [19]. This has been attributed to swallowing of infected pulmonary secretions [20, 21].

Shedding has been extensively assessed only in Eurasian badgers (*Meles meles*) in natural and experimental infections. It was shown by bacteriological culture to occur by the fecal, urinary, pulmonary and oronasal routes and wound discharges in 25–50% of infected animals [12, 20, 22–24]. In this species a “super-shedder” state has been described encompassing those badgers where MTC shedding is detected by culture persistently or by more than one route, in contrast to the standard, intermittent shedders [23]. “Super-shedders” have been hypothesized to occur also in the wild boar [25] but evidence to support this supposition was lacking.

Knowledge of the excretion routes is crucial to improve the control strategies to reduce bTB in wildlife. Determining which excretion route(s) is(are) more prevalent is fundamental to define the likelihood of interspecific transmission as fecal shedding tends to promote indirect transmission through contamination of the environment, while oronasal shedding, in addition to environmental contamination, allows also an easier direct transmission by aerosols, usually involving conspecifics [2, 26]. Although the anatomical location of lesions *per se* provides some information on the potential routes of transmission, this indirect association needs to be interpreted carefully as abdominal lesions can be caused by swallowing of infected pulmonary secretions or hematogenous spread of infection [2, 20, 26].

As the understanding of the bTB excretion routes and MTC excretion doses is critical for defining the best control strategies for wild reservoirs, it is surprising that so little solid data is available on this subject [1]. The aim of this study was thus to determine the MTC excretion routes and concentration of MTC in the biological samples from the potential transmission routes. This was performed by molecular biology methods using samples from naturally infected hunter-harvested wild boar and red deer, for which the bTB status was defined.

Among several protocols tested, a nested PCR was selected as it revealed the highest sensitivity for the MTC molecular detection. Besides detection of MTC shedding it is of utmost importance to quantify excretion. Since DNA present in samples is not quantifiable by nested PCR protocols, we combined this with the Most Probable Number (MPN) method [27]. The MPN is an established and well documented technique to obtain estimates of microbial concentrations from binomial data [27].

## Materials and methods

### Study design

In order to investigate the MTC excretion routes from naturally infected wild ungulates we collected, from hunter-harvested wild boar (*n* = 116) and red deer (*n* = 62), the head and distal third of the neck (66 wild boar and 33 deer), lungs and proximal third of the trachea (66 wild boar and 54 deer), feces from the rectum (93 wild boar and 41 deer) and urine samples from the urinary bladder (3 wild boar and 1 red deer). We obtained bronchial-alveolar lavages (BAL) by aseptically pouring 100 mL of sterile water into the trachea, inverting the lungs and collecting the washes from the trachea, and also oronasal lavages (ONL) by pouring 100 mL of sterile water into the pharynx and collecting the washes from the nose and mouth. All samples were stored at −20 °C until processing, up to 12 weeks post-collection.

Infection status of hunter-harvested wild boar and red deer was assessed by gross pathology, PCR in the lymph nodes with macroscopic lesions and bacteriological culture following protocols previously described [28]. Taking into account these results, the animals were categorized into the following groups: (1) bTB-confirmed—macroscopic lesions detected and MTC demonstrated in tissues by culture or molecular methods (56 wild boar and 43 red deer); (2) bTB-suspected—macroscopic lesions detected, bacteriological culture and PCR-negative in tissues (21 wild boar and 15 red deer); (3) bTB-free—negative for gross pathology, PCR and culture in tissues and collected in regions where bTB has not been detected in wildlife despite surveillance (31 wild boar and 4 red deer).

Regions with known bTB infection status in wild ungulate populations were identified from published results [29] and consisted of the following Portuguese counties: Idanha a Nova (centroid coordinates, utm wgs84: 661408, 4418202), Castelo de Vide (629710, 4369235), Moura (650202, 4221830) and Mértola (614207, 4167236).

### DNA extraction protocol

40 mL of lavages (BAL or ONL) or urine were centrifuged at 2566 *g* for 30 min (Heraeus Multifuge 3SR Plus, ThermoFisher Scientific, Waltham, MA, USA), after which most of the supernatant was discarded and 0.5 mL aliquots of the sediment/supernatant interface were collected for DNA extraction. 15 g of fecal material were agitated overnight at 150 rpm at 8 °C in an incubation shaker (Multitron II, Infors AG, Bottmingen, Switzerland) in order to homogenize the sample. After resting for 2 h at room temperature, 14 mL of the supernatant/sediment interface were collected and processed as previously described for lavages and urine samples.

For lavages, DNA extraction was performed by a standard phenol–chloroform protocol. Briefly, 55 µL of 10 × TEN buffer and 0.25 mL phenol were added to 0.5 mL of sample in a 2 mL screw-cap conical tube containing 100 µL of 0.1 mm zirconia/silica beads (Biospec Products, Bartlesville, OK, USA). The mixture was subjected to 2 cycles of 30 s agitation at 5 m/s in a FastPrep 24 (MP Biomedicals, Santa Ana, CA, USA), after which 0.25 mL chloroform were added and gently agitated for 60 s, followed by 5 min centrifugation at 16 627 *g* at 4 °C. 500 µL of the aqueous phase was then transferred to a new tube and an equal volume of chloroform added, mixed by gentle agitation for 60 s and again centrifuged for 5 min at 16 627 *g* at 4 °C. 300 µL of the aqueous phase were then transferred to a new tube and 40 µL of sodium acetate and 800 µL absolute EtHO were added and this mix was left to rest for 2 h at room temperature, followed by 10 min centrifugation at 19 283 *g* at 4 °C. The supernatant was discarded and the pellet washed with 70% EtHO, centrifuged for 5 min at 16 627 *g* at 4 °C, the supernatant again discarded and the pellet suspended in 50 µL of TE buffer.

For fecal and urine samples, DNA extraction was performed using a slight modification of the protocol by Griffiths et al. [30]. The differences to the abovementioned phenol–chloroform extraction protocol were: 0.5 mL of 5% hexadecyltrimethylammonium bromide buffer were used instead of 10× TEN buffer and DNA was precipitated by the addition of 400 µL of 30% PEG 6000 solution in 1.6 M NaCl_2_ and kept at room temperature for 2 h.

Quantification and purity assessment of DNA was performed using NanoDrop (ThermoScientific, Wilmington, DE, USA). DNA extraction negative controls were included at a rate of 1 for every 6 samples.

### Molecular detection

As screening test for MTC DNA, a modification of the nested PCR protocol targeting IS6110 described by Soo et al. [31] was used, including the same set of internal and external primers (external forward: 5′ CGTGAGGGCATCGAGGTGGC 3′, external reverse: 5′ GCGTAGGCGTCGGTGACAAA 3′, internal forward: 5′ CTCGTCCAGCGCCGCTTCGG 3′, internal reverse: 5′ GCGTCGGTGACAAAGGCCAC 3′). Briefly, 250 ng DNA were added to a solution of 7.5 μL of NZYTech Green Master Mix (NZYTech, Lisbon, Portugal), containing 1.5 U Taq polymerase, 1.5 mM MgCl_2_, 1 μL of each primer at 20 mM and 5% DMSO, in a final volume of 25 μL. For the internal PCR 1 μL of the products of the external PCR was used as template. External PCR mix were submitted to the following PCR protocol: initial denaturation at 94 °C for 5 min, followed by 26 cycles of 94 °C for 30 s, annealing at 64 °C for 15 s and extension at 72 °C for 30 s, with a final extension step of 72 °C for 3 min. Internal PCR mix were submitted to the same protocol, except that 30 cycles were used. Negative controls were included in all PCR at a rate of 1 for every 3 samples.

As an external control for PCR inhibition, every sample negative for MTC DNA was inoculated with 7 × 10^4^ copies of a PCN1 construct inserted in a pGEM plasmid and subjected to a standard PCR using the primers forward: 5′ ATACGACTCACTATAGGGCG 3′, reverse: 5′ GGTGACACTATAGAATACTC 3′. Briefly, 0.25 pg of pGEM PCN1 DNA and 250 ng of DNA extracted from the biological samples were added to a solution of 12.5 μL of NZYTech Green Master Mix, containing 2.5 U Taq polymerase, 3.0 mM MgCl_2_, 1 μL of each primer at 20 mM and 5% DMSO, in a final volume of 25 μL. This mix was submitted to the following PCR protocol: initial denaturation at 94 °C for 5 min, followed by 45 cycles of 94 °C for 30 s, annealing at 52 °C for 30 s and extension at 72 °C for 30 s, with a final extension step of 72 °C for 3 min. Inhibition was detected in 44/209 samples, which were then diluted 1:2 or 1:4 until inhibition disappeared. In all but 18 samples PCR inhibition was avoided using these method; these 18 samples from fecal extracts (*n* = 16), ONL and BAL (*n* = 1 each) were removed from the analysis.

PCR products were visualized by electrophoresis in 2% agarose gel with GreenSafe Premium (NZYTech, Lisbon, Portugal) and photographed under UV light with Alpha Imager (Alpha Innotech Corporation, San Leandro, CA, USA). The preparation of the nested PCR master mixes took place in a room not used for other work with MTC and physically separate from the rooms where the addition of the DNA templates was performed. Negative controls were included at a rate of 1 for every 3 samples.

### Bacteriological culture

In a restricted set of samples (18 ONL, 13 BAL and 12 fecal samples from 5 wild boar and 7 red deer) bacteriological culture for *M. bovis* detection was performed. Briefly, 15 mL of lavages or 15 g of feces were decontaminated for 2 h with 30 mL of 0.75% hexa-decyl-pyridinium chloride solution, after which they were centrifuged at 2566*g* for 30 min; most supernatant was discarded and 0.25 mL aliquots of the sediment-supernatant inoculated in Coletsos medium (2 tubes for each sample) (BioMerieux, Marcy l’Étoile, France). The inoculated tubes were incubated at 37 °C for 15 weeks, checked weekly for any growth suspected to be MTC, which was then re-inoculated again in Coletsos medium. Isolates were identified by PCR for a panel of selected genes: 16S RNA, IS1081, Rv3120 and Rv1510 following the protocol by Huard et al. [32]. Briefly, 250 ng DNA were added to a solution of 6.5 μL of NZYTech Green Master Mix (NZYTech, Lisbon, Portugal), containing 1.3 U Taq polymerase, 1.5 mM MgCl_2_, 1 μL of each primer at 20 mM and 5% DMSO, in a final volume of 25 μL. This mix was submitted to the following PCR protocol: denaturation at 94 °C for 5 min, 35 cycles at 94 °C for 1 min, annealing at 60 °C for 1 min and extension at 72 °C for 1 min, with a final extension step of 72 °C for 10 min.

### Most probable number

MTC concentration was estimated using the method Most Probable Number (MPN) [27] based on positive/negative nested PCR data on serial dilutions of DNA. Briefly, serial tenfold dilutions of MTC-positive DNA samples were submitted to the previously described nested PCR protocol targeting IS6110 [31]. Undiluted DNA was assayed in triplicate, 1:10, 1:10^2^, 1:10^3^ and 1:10^4^ DNA were assayed 1–2 times. The dilution at which no detection begins to occur indicates that the DNA has been diluted so much as to be absent and is used to estimate the original concentration. The software MPN Calculator Build 23 [33] was used to compute the MTC DNA concentration.

Samples of excretion routes obtained from animals negative for bTB and where no MTC DNA was detected were inoculated with twofold decreasing concentrations of *M. bovis* bacillus Calmette-Guérin (BCG) strain Pasteur, determined by colony-forming units (CFU). Negative controls were included in each assay, consisting of the same substrate inoculated with the same volume of sterile water. After seeding, the samples were manually agitated to homogenize the mycobacterial distribution and subjected to the molecular detection techniques previously described. The 100% limit of detection (LD100) was determined after repeating 7 times the molecular detection protocols in the inoculated samples.

**Figure 1:**
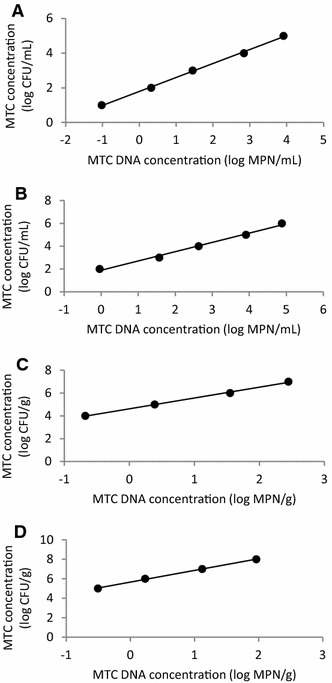
**Calibration line for determining MTC concentration using estimates of MTC DNA concentration.** Least squares linear regression between MTC DNA concentration estimated by the MPN (as log MPN/g or mL) and inoculated BCG concentration (as log CFU/g or mL) in oro-nasal lavages (**A**), bronchial-alveolar lavages (**B**), wild boar fecal samples (**C**) and red deer fecal samples (**D**). R^2^ is 0.999 and slope 0.806 for ONL, 0.993 and 0.815 for BAL, 0.998 and 0.497 for wild boar feces and 0.999 and 1.203 for red deer feces.

Calibration lines were calculated by applying the MPN technique to inoculated biological samples. BCG concentrations and MPN estimates were log transformed and their least squares linear relation was calculated and used to convert MPN estimates of MTC DNA concentration to MTC concentration in CFU/g or mL. For ONL and BAL the relation between inoculated BCG concentration and MPN estimates was linear over 5 log, for wild boar and red deer feces over 4 log (Figure 1).

### Statistical analysis

Fisher’s exact test, Mann–Whitney U test and binary logistic regression were performed in IBM SPSS Statistics (SPSS, Chicago, ILL, USA); graphics were produced in Excel 2007 (Microsoft, Redmond, WA, USA); and confidence intervals for the positivity rates were calculated using VassarStats [34].

## Results

### MTC excretion was detected in most bTB-confirmed or -suspected ungulates

In order to characterize MTC excretion the first step was to determine the limits of detection of the techniques used. For ONL and BAL the LD100 was 5 × 10^2^ CFU/mL. For wild boar feces the LD100 was 5 × 10^4^ CFU/g, while for red deer feces the LD100 was 4 × 10^6^ CFU/g.

Overall, MTC DNA was detected in 82/173 wild boar samples and 61/118 red deer samples from bTB-confirmed or suspected animals (Table 1). MTC DNA was not detected in any of 43 samples from negative controls, i.e. animals from bTB-negative regions and lesion- and culture-negative: red deer BAL (*n* = 4) and ONL (*n* = 2) and wild boar BAL (*n* = 10) and feces (*n* = 27). Considering only those bTB-confirmed or suspected ungulates for which all three types of biological samples (ONL, BAL and feces) were available, MTC DNA was detected in at least one biological sample in 31/39 wild boar (79.5%, CI_95_ 64.5–89.2) and 13/14 red deer (92.9%, CI_95_ 68.5–98.7). Moreover, MTC DNA was amplified in all three types of biological samples in 5/39 wild boar (12.8%, CI_95_ 5.6–26.7) and 2/14 red deer (14.3%, CI_95_ 4.0–40.0). No statistically significant differences in MTC detection rate were found between species. MTC excretion was detected at approximately the same rates in bTB-confirmed or suspected groups, with the exception of red deer BAL, where MTC DNA was amplified significantly more often in bTB-confirmed than in bTB-suspected deer (*p* = 0.004, Fisher’s exact test) (Table 1). *M. bovis* was isolated by culture from the feces of one infected wild boar out of 7 samples that were not overgrown by other microorganisms (3 ONL, 2 BAL and 2 feces).

**Table 1:** Proportion of excretion samples positive for MTC DNA.

Host species	Excretion route	bTB-confirmed status				bTB-suspected status			
		No. tested	MTC-positive samples			No. tested	MTC-positive samples		
			no.	%	CI_95_ (%)		no.	%	CI_95_ (%)
Wild boar	Oronasal	47	26	55.3	41.3–68.6	17	7	41.2	21.6–64.0
	Bronchial-alveolar	39	25	64.1	4.48–77.3	17	10	58.8	36.0–78.4
	Fecal	34	9	26.5	14.6–43.1	17	3	17.7	6.2–41.0
	Urinary	3	2	66.7	20.8–93.9	0			
	Total	123	62	50.4	41.7–59.1	51	20	39.2	27.0–52.9
Red deer	Oronasal	22	10	45.5	26.9–65.3	8	5	62.5	30.6–86.3
	Bronchial-alveolar	36	29	80.6	65.0–90.3	12	4	33.3**	13.8–60.9
	Fecal	30	9	30.0	16.7–47.9	9	3	33.3	12.1–64.6
	Urinary	0				1	1	100	20.7–100
	Total	88	48	54.6	44.2–64.5	30	13	43.3	27.4–60.8

Proportion of MTC positive biological samples by species, excretion route and infection status, with confidence intervals and statistically significant differences between infection status highlighted (Fisher’s exact test).

** *p* < 0.01.

No seasonal, age or gender differences were found for the other types of biological samples or species. In a binary logistic regression analysis with MTC detection as dependent variable and species, gender, age, season, bTB status and type of biological sample as independent variables, the only factor affecting the proportion of MTC DNA positive samples was the type of biological sample, with fecal shedding being detected less often than oronasal or bronchial-alveolar shedding in both species (*p* < 0.001).

### A proportion of the infected ungulates excrete large concentrations of MTC DNA by several routes

**Figure 2:**
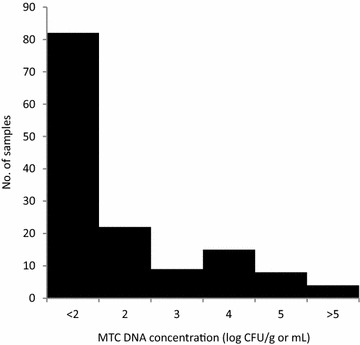
**Distribution of the estimated** ***Mycobacterium tuberculosis*** **complex concentrations.** Distribution of the MTC DNA concentrations (as log CFU/g or mL) in the oro-nasal and bronchial-alveolar lavages and fecal samples in which MTC DNA was amplified (*n* = 140), both host species combined. MTC DNA concentrations were estimated based on the calibration line between log-transformed inoculated BCG concentrations (CFU/g or mL) and MPN concentration estimates (MPN/g or mL).

MTC DNA concentration in positive samples revealed a bimodal pattern separated at the concentration 10^3^ CFU/g or mL (Figure 2). The ungulates with >10^3^ CFU/g or mL in at least 1 sample were 14 wild boar and 22 red deer. Considering only those ungulates for which all three biological samples (ONL, BAL and feces) were available for this study, 28.2% of wild boar (CI_95_ 16.6–43.8) and 35.7% of red deer (CI_95_ 16.3–61.2) had at least one excretion route with >10^3^ CFU/g or mL.

The proportion of male wild boar with MTC DNA concentrations >10^3^ CFU/g or mL was higher than that of females (42.9 vs 21.4%), while in red deer the opposite was true (0% for males vs 36.4% for females), although these differences were not statistically significant (Table 2).

**Table 2:** Characterization of super-shedders.

Host species	Gender	Age	bTB status	MTC DNA concentration (CFU/g or mL)		
				Feces	Bronchial-alveolar lavages	Oro-nasal lavages
Red deer	Female	Adult	Suspected	Neg	70	4.3 × 10^3^
	Female	Adult	Confirmed	9.9 × 10^4^	248	4.3 × 10^3^
	Female	Adult	Confirmed	Neg	<10	4.3 × 10^3^
	Female	Adult	Confirmed	9.9 × 10^4^	1.9 × 10^4^	99
Wild boar	n.a.	n.a.	Confirmed	Neg	93	4.3 × 10^3^
	n.a.	Adult	Confirmed	1.3 × 10^4^	<10	557
	Female	Subadult	Confirmed	3.2 × 10^4^	14	557
	Male	Adult	Confirmed	6.0 × 10^6^	<10	178
	Female	Adult	Confirmed	1.3 × 10^4^	<10	557
	n.a.	n.a.	Confirmed	Neg	<10	9.9 × 10^3^
	Female	Adult	Suspected	1.0 × 10^5^	Neg	178
	n.a.	Subadult	Confirmed	1.0 × 10^5^	Neg	Neg
	n.a.	Adult	Confirmed	1.9 × 10^5^	Neg	679
	Male	Adult	Suspected	3.2 × 10^4^	<10	Neg
	Male	n.a.	Confirmed	1.3 × 10^4^	Neg	Neg

Species, age, gender, infection status and MTC DNA concentration in samples from the ungulates with at least one sample with MTC DNA concentration >10^3^ CFU/g or mL (highlighted in bold) out of 3 tested samples. MTC DNA concentrations were estimated based on the calibration line between log-transformed inoculated BCG concentrations (CFU/g or mL) and MPN concentration estimates (MPN/g or mL).

n.a not available.

In a binary logistic regression analysis for those samples where MTC DNA was amplified (80 wild boar and 60 red deer samples), with MTC DNA concentration classified as lower or higher than 10^3^ CFU/g or mL as dependent variable and species, age, gender, season, bTB status and type of biological sample as independent variables, host species was significantly related with concentration (*p* < 0.01). Red deer showed a tendency for MTC DNA concentrations >10^3^ CFU/g or mL (OR = 11.8, CI_95_ 2.3–60.2).

Red deer with at least one sample with >10^3^ CFU/g or mL showed significantly higher MTC DNA concentrations in BAL compared to wild boar (*p* = 0.05, Mann–Whitney U). When considering only ungulates with <10^3^ CFU/g or mL in all samples tested no differences were found between species. Super-shedder ungulates excreted significantly higher concentrations of MTC than standard shedders as detected in lavages (*p* < 0.01 for both species, Mann–Whitney U) (Table 3).

**Table 3:** Average MTC DNA concentration in excretion samples from wild ungulates with ONL, BAL and fecal samples tested.

Host species	Excretion route	Average MTC DNA concentration (CFU/g or mL)			
		No.	Super-shedders	No.	Standard shedders
Wild boar	Oronasal	11	1.5 × 10^3^	20	182.2
	Bronchial-alveolar	11	15.2	20	22.8
	Fecal	11	5.9 × 10^5^	20	0
Red deer	Oronasal	5	2.6 × 10^3^	7	83.7
	Bronchial-alveolar	5	3.8 × 10^3^*	7	12.9
	Fecal	5	9.8 × 10^4^	7	0

Average MTC DNA concentration by host species and excretion route, including ungulates with >10^3^ CFU/g or mL in at least one sample (super shedders) or only those with <10^3^ CFU/g or mL in all samples (standard shedders). Statistically significant differences between species are highlighted (Mann–Whitney U). MTC DNA concentrations were estimated based on the calibration line between log-transformed inoculated BCG concentrations (CFU/g or mL) and MPN concentration estimates (MPN/g or mL).

* *p* = 0.05.

## Discussion

We provide here evidence and quantify, for the first time, MTC excretion by several routes from naturally infected wild boar and red deer. MTC DNA was detected in all types of biological samples investigated (oronasal and bronchial-alveolar lavages, feces and urine). In 80% of all naturally-infected wild ungulates for which ONL, BAL and feces were available we amplified MTC DNA in at least one sample. This proportion of shedders is higher than reported previously for red deer [15]. This discrepancy might be due to a lower sensitivity of bacteriological culture from swabs performed in the previous study, compared to the molecular detection in lavages and fecal samples used here [24, 35]. This proportion of shedders is also much higher than the one reported for badgers [23] and the difference could be due to the same methodological factors or to differences in bTB pathology between species [10]. In fact, there is evidence that most excretor badgers could be those in advanced, terminal stages of bTB [20, 36] whereas in wild ungulates excretion seems to occur intermittently from early stages of disease [18].

Our results may have been influenced by the fact that bTB lesions could perforate either during hunting or during evisceration of the carcasses, releasing previously encapsulated MTC into the bronchial-alveolar compartment or into the oronasal cavity. This could have led to distorted rates of excretion and estimates of MTC DNA concentration in some samples. Nevertheless the differences found on excretion in our sample associated with known host disease determinants such as species and gender cannot be explained by these methodological issues and should reveal true biological processes. Also our molecular detection protocol targets all MTC species, which could lead to the detection of other mycobacteria not responsible for bTB. Among these, *M. microti* has been reported to infect wild boar [37], although this has never been reported in Iberian Peninsula. Nevertheless all our negative control samples yielded no amplification of MTC DNA, which leads to the assumption that *M. microti* excretion does not occur, at least to a significant extent, in our sample. Also freezing of the biological samples at −20 °C could have affected the viability of mycobacteria and so contributed to the low success of the bacteriological culture. Nevertheless Tessema et al. [38] found no effect of freezing sputum samples at −20 °C on the success of *M. tuberculosis* bacteriological culture.

For the first time we report evidence of the occurrence in wild ungulates of a class of infected hosts that fit into the definition of super-shedders. Super-shedders have been described in the Eurasian badger as those in which MTC excretion is detected consistently trough time or by several routes [23]. Although our study design is cross-sectional, so we do not assess the temporal dimension, we found that a proportion of infected ungulates (28.2% of wild boar and 35.7% of red deer) excrete MTC at large concentrations by at least one route. Also shedding was detected to occur by all three routes analyzed in a proportion of the infected ungulates (12.8–14.3% of wild boar and red deer, respectively). Moreover in 6 out of 15 super-shedders for which all 3 routes were available, MTC DNA was amplified in all routes. Furthermore, 13 out of 15 super-shedders had at least another MTC DNA-positive excretion route (in two cases also with MTC concentrations >10^3^ CFU/g or mL) (Table 2). This means that a large proportion of the ungulates excreting large concentrations of MTC by one route, are in fact excreting by several routes, further supporting their classification as super-shedders.

Although MTC DNA concentration was not directly measured on the excretion samples, our estimates suggest that a super-shedder ungulate sheds on average >10^5^ CFU/g or mL through all routes combined (Table 3). Given the known infectious doses for cattle (10^2^–10^3^ CFU by inhalatory route and 5 × 10^3^ CFU by oral route), red deer (10–5 × 10^2^ CFU orally) and wild boar (10^4^ CFU oropharingeal route) [13, 14, 17, 39], the estimated quantity of MTC excreted by a super-shedder wild boar or red deer would be sufficient to infect these hosts. Also MTC DNA concentrations excreted by super-shedders are at least one order of magnitude higher than those excreted by standard shedders. This supports the super-shedder subset of the infected population of wild ungulates has having a disproportionally large role in the transmission and maintenance of bTB in multi-host pathogen systems.

The existence of super-shedders in bTB-infected wild ungulates has implications for the design of control programs in these species. In fact, the removal of super-shedders from the population could reduce drastically the horizontal transmission and environmental contamination with MTC, which should lead to a decline on bTB incidence. The elimination of super-shedders could be accomplished by selective culling, but requires the previous identification of correlates of super-excretion, allowing targeting these animals in culling actions. Red deer females and wild boar males tend to be overrepresented in the super-shedder subset, although the differences are not statistically significant. Further studies are needed to characterize the super-shedder subset of the infected population of both species.

A future approach to reduce super-shedders would be to vaccinate against bTB with live or inactivated oral vaccines that are presently under development and validation for use in free-ranging wild ungulate populations, namely the wild boar [14, 40–42] and the white-tailed deer [43, 44]. Although these vaccines do not protect from infection or disease they diminish the severity of lesions and mycobacterial load in tissues [e.g. 40, 42] and so could potentially hamper the build-up to a super-shedder status.

Excretion was detected in a significantly lower proportion of fecal samples compared to oronasal or bronchial-alveolar samples in both host species. Abdominal lesions are detected less often compared to thoracic and cephalic lesions in the wild boar [8] but not in the red deer [9]. Nevertheless, cephalic and thoracic lesions can also give rise to fecal excretion by swallowing oral and pulmonary secretions [21]. Another possible explanation is the higher detection limit of our protocol when applied to fecal samples compared to lavages, which could give rise to a greater proportion of false negative results in fecal samples. In fact while the LD100 was equal in both lavages, it was 100–10 000× greater in feces, which explains why MTC DNA was amplified in fecal extracts only from super-shedders, as the standard shedders by this route would not be detected with the protocol we describe. The DNA extraction protocol by Griffiths et al. [30] was adopted for fecal samples because it allows controlling co-extracted PCR inhibitors in the fecal material [45], which were found to hamper PCR reactions in preliminary assays when the standard phenol–chloroform method was used.

Although we could only collect a limited number of urine samples due to the processing of hunted ungulates carcasses, which usually leads to rupture of the urinary bladder, it was surprising to find such a high proportion of shedders by this route (3 out of 4). In fact, the reported prevalence of kidney lesions is low in both wild boar and red deer [8, 9], which may be explained by the difficulty in detecting bTB lesions in organs with a large parenchyma or to the presence of microscopic lesions often missed by gross pathology [10, 20]. These results highlight that further studies on the urinary excretion of MTC and prevalence of kidney bTB lesions in wild ungulates are needed.

In super-shedder ungulates in our sample, MTC DNA concentrations in bronchial-alveolar lavages were significantly higher in red deer than in wild boar. This is expected given the structure of the lesions in each species, with red deer usually showing abscesses often located in the lungs and moderate numbers of acid-fact bacilli, while wild boar tend to show caseocalcareous lesions predominantly located in lymph nodes with a small number of acid-fast bacilli [5, 8, 9]. In wild boar the biological samples with higher average MTC DNA concentration (oronasal lavages) coincide with the most frequent anatomical location of bTB lesions (cephalic lymph nodes) [8].

On this article we report the detection of MTC excretion in 80% of bTB-naturally-infected wild boar and red deer. For the first time we provide evidence for the existence of a proportion of super-shedders within the naturally infected population of these host species. These super-shedders are responsible for a disproportionately large amount of MTC excretion from infected wild ungulates. MTC DNA concentrations greater than the minimum infective doses for cattle, red deer or wild boar are present in excretion routes from both species. These results have implications for the design of control programs in multi-host pathogen systems where these species are maintenance hosts for bTB.
